# Lipase-Catalyzed Synthesis of Structured Fatty Acids Enriched with Medium and Long-Chain n-3 Fatty Acids via Solvent-Free Transesterification of Skipjack Tuna Eyeball Oil and Commercial Butterfat

**DOI:** 10.3390/foods13020347

**Published:** 2024-01-22

**Authors:** Khurshid Ahmed Baloch, Umesh Patil, Khamtorn Pudtikajorn, Ebtihal Khojah, Mohammad Fikry, Soottawat Benjakul

**Affiliations:** 1 International Center of Excellence in Seafood Science and Innovation (ICE-SSI), Faculty of Agro-Industry, Prince of Songkla University, Hat Yai 90110, Songkhla, Thailand; khurshidbaloch555@gmail.com (K.A.B.); umesh.p@psu.ac.th (U.P.); khamtorn.p@gmail.com (K.P.); moh.eltahlawy@fagr.bu.edu.eg (M.F.); 2 Department of Food Science and Nutrition, College of Sciences, Taif University, P.O. 11099, Taif 21944, Saudi Arabia; 3 Department of Agricultural and Biosystems Engineering, Faculty of Agriculture, Benha University, Moshtohor, Toukh 13736, Egypt; 4 Department of Food and Nutrition, Kyung Hee University, Seoul 02447, Republic of Korea

**Keywords:** structured lipid, infant formula milk, medium-chain fatty acids, unsaturated fatty acid, human milk fat, Asian seabass lipase, commercial butterfat

## Abstract

Human milk lipids generally have the maximum long-chain fatty acids at the sn-2 position of the glycerol backbone. This positioning makes them more digestible than long-chain fatty acids located at the sn-1, 3 positions. These unique fatty acid distributions are not found elsewhere in nature. When lactation is insufficient, infant formula milk has been used as a substitute. However, the distribution of most fatty acids ininfant formula milk is still different from human milk. Therefore, structured lipids were produced by the redistribution of medium-chain fatty acids from commercial butterfat (CBF) and n-3 and n-6 long-chain fatty acids from skipjack tuna eyeball oil (STEO). Redistribution was carried out via transesterification facilitated by Asian seabass liver lipase (ASL-L). Under the optimum conditions including a CBF/STEO ratio (3:1), transesterification time (60 h), and ASL-L unit (250 U), the newly formed modified-STEO (M-STEO) contained 93.56% triacylglycerol (TAG), 0.31% diacylglycerol (DAG), and 0.02% monoacylglycerol (MAG). The incorporated medium-chain fatty acids accounted for 18.2% of M-STEO, whereas ASL-L could incorporate 40% of n-3 fatty acids and 25–30% palmitic acid in M-STEO. The ^1^H NMRA and ^13^CNMR results revealed that the major saturated fatty acid (palmitic acid) and unsaturated fatty acids (DHA and EPA) were distributed at the sn-2 position of the TAGs in M-STEO. Thus, M-STEO enriched with medium-chain fatty acids and n-3 fatty acids positioned at the sn-2 position of TAGs can be a potential substitute for human milk fatty acids in infant formula milk (IFM).

## 1. Introduction

Lipids are important biological macromolecules that regulate hormones, transmit nerve impulses, provide passages for fat-soluble nutrients, and preserve excessive energy in the body [1]. The physicochemical, nutritional, and functional properties of lipids depend on the length of the carbon chain, the number of double bonds and their positions, and the location of fatty acids in the glycerol backbone [2]. Fatty acids are categorized as long-chain fatty acids (LCFAs), medium-chain fatty acids (MCFAs), and short-chain fatty acids (SCFAs) [3]. The consumption of LCFAs located at positions sn-1 and 3 raises the percentage of calories, compared to LCFAs at the sn-2 position. The consumption of lipids with LCFAs at the sn-1 and 3 positions are strongly linked to being overweight or obese [2]. SCFAs and MCFAs show better digestibility and serve as biological energy and contribute to newborns feeling full for longer periods [3]. More importantly, these fatty acids are not adipogenic and are immediately absorbed into the bloodstream after consumption [3]. On the other hand, triacylglycerol rich in MCFAs can help in losing or controlling body weight. MCFAs are fatty acids possessing 6–12 carbon chains. Naturally, they are available in milk fat and coconut oil [4].

Long-chain unsaturated fatty acids are important for the growth of the central nervous system in infants and the cardiovascular system in adults [5,6]. The development of infant organs and biological systems depends entirely on the nutrients present in human milk. The optimal development of the baby depends on the presence of essential fatty acids in human milk [7]. Comparing the milk of different mammals, such as cow, goat, and porcine milk, human milk has the highest concentration of unsaturated fatty acids (UFAs) [8]. A significant portion of the world’s population struggles frequently with lactation insufficiency and a scarcity of essential fatty acids in their daily consumption. During the aforementioned period, infants must be fed with infant formula milk (IFM). Nevertheless, IFM is typically made from either cow or goat milks, which are lacking in vital UFAs found in human milk [9]. As a result, the development of structured human milk fatty acid substitution (HMFAS) has gained increasing interest. Small-, medium-, and long-chain unsaturated fatty acids are enzymatically incorporated into various oils or lipids to produce HMFAS [10].

Through the biochemical engineering and modification of lipids, structured lipids (sLPs) can be produced, thereby augmenting both their nutritional and functional characteristics. The production of sLPs can be accomplished via interesterification either with chemical means such as acid/base chemical catalysts or by mediating the reaction with lipase [2]. When compared to chemical interesterification, enzymatic interesterification has more advantages such as excellent selectivity, mild reaction conditions, less unwanted by-products, less waste, and ease of product recovery [2]. Enzymatic interesterification can be performed with four specific mechanisms such as acidolysis, alcoholysis, glycerolysis, and transesterification [2]. Transesterification is one of the commonly used forms of interesterification. When transesterification takes place, the ester bonds between the fatty acids and glycerol backbone are cleaved, thus liberating fatty acids. The liberated fatty acids are then randomly distributed in a fatty acid pool where they can reposition themselves either on the same or different glycerol backbones [11].

Seafood industry waste is abundant in biologically important healthy biomolecules such as enzymes, bio-calcium, n-3 fatty acids, and collagen [12]. To valorize the waste produced in the processing of the seafood industry, various processing leftovers such as fish viscera, fish skin, fish bones, and fish heads have been exploited to obtain enzymes and other proteins, collagen, bio-calcium, and eyeball oil [13,14,15,16]. Fish viscera contain various hydrolytic enzymes including lipases [14]. Fish lipases are more specific toward polyunsaturated fatty acids because they naturally deal with UFAs [17,18]. The fish eyeball contains oil rich in polyunsaturated fatty acids that can be modified and converted into more valuable products [19]. Butter is a fat-rich semi-solid substance produced from milk, commonly found as an oil-in-water emulsion. Commercial butterfat (CBF) is consisted of more than 96% triacylglycerols (TAGs) [20]. Medium- and long-chain saturated FAs in most of the milk fat account for approximately 50–60% of all FAs. Additionally, CBF is also abundant in short-chain FAs (C4:0–C8:0) and medium-chain FAs (C10:0–C14:0) [21].

This study was performed to explore the ability of the Asian seabass liver lipase (ASL-L) to produce sLPs from the skipjack tuna eyeball oil (STEO) and CBF. STEO is rich in n-3 fatty acids and CBF is rich in medium-chain fatty acids. Nonetheless, ASL-L has never been applied to produce structured lipids and lipases from marine sources, which would be more specific to the n-3 and n-6 long-chain unsaturated fatty acids [17,18]. Therefore, ASL-L was used to perform solvent-free transesterification of STEO and CBF to incorporate low-caloric medium-chain fatty acids onto the STEO glycerol backbone, thus producing a modified STEO (M-STEO) enriched with medium-chain fatty acids and n-3 fatty acids, having a similar fatty acid composition to that of human milk. M-STEO can further be used as an infant formula milk (IFM) containing fatty acids closely related to those found in human milk.

## 2. Materials and Methods

### 2.1. Chemicals

The chemicals used in this study were of analytical grade. Commercial butterfat (Allowrie Foods Australia, Sydney, Australia) was purchased from a local supermarket and used as CBF. *p*-nitrophenyl palmitate (*p*-NPP) was acquired from Sigma-Aldrich (St. Louis, MO, USA).

The skipjack tuna eyeball oil (STEO) was extracted following the method of Pudtikajorn et al. [15]. Briefly, tuna eyeballs were chopped into small pieces using a chopper (Electrolux model EFP7804S, Stockholm, Sweden) for 1 min. The disintegrated eyeballs were mixed with distilled water (1:1, *w*/*v*) and transferred into 250 mL airtight bottles (DWK Life Sciences GmbH, Mainz, Germany) and autoclaved (TOMY model SX-500E, Tokyo, Japan) at 121 °C for 60 min. The bottles were cooled down using running tap water. The mixtures were centrifuged at 3000× *g* at room temperature for 5 min. Cheesecloth was used to remove the upper floating phase and the emulsion was subsequently subjected to centrifugation at 10,000× *g* at room temperature for 10 min. The oil phase on the top was removed with a separating funnel, 20% of anhydrous sodium sulfate was added, and this was named as skipjack tuna eyeball oil (STEO).

### 2.2. Asian Seabass Liver Lipase (ASL-L) Powder Production

Asian seabass viscera (*Lates calcarifer*) (5 kg) were acquired from a nearby fresh market in Hat Yai, Songkhla, Thailand. Seabass liver powder was prepared as described by Baloch et al. [22]. Briefly, freshly removed viscera were placed in polyethylene bags and transported in ice to the laboratory within 30–40 min. The livers were freshly separated from the samples and adipose tissues were removed. Livers were cut into small pieces and blended in liquid nitrogen with a blender. The blended mixture was defatted by adding chilled acetone at 1:3 (*w*/*v*) and homogenized at 15,000 rpm for 5 min using an IKA Labortechnik homogenizer (Selangor, Malaysia). The mixture was then agitated using an overhead stirrer (Model RW20.n, IKA-Werke Gmb H&CO. KG, Staufen, Germany) for 40 min at 300 rpm and 4 °C and vacuum-filtered with Whatman paper No. 4. The defatted samples were air-dried overnight. The dried samples of the so-called liver powder (LP) were kept in zip-lock bags at −40 °C until further use.

### 2.3. Extraction of Asian Seabass Liver Lipase (ASL-L)

Asian seabass liver lipase (ASL-L) was extracted using an extraction buffer (25 mM Tris–HCl, pH 8.0 containing 1 mM CaCl_2_) [14]. Firstly, LP was dispersed in the extraction buffer at a ratio of 1:9 (*w*/*v*) and thoroughly stirred for 40 min at 4 °C with an overhead stirrer (Model RW20.n, IKA-Werke Gmb H&CO. KG, Staufen, Germany) at 300 rpm. Afterward, the samples were centrifuged at 10,000× *g* and 4 °C for 30 min. The supernatant was filtered with Whatman paper No. 4 to remove the remaining fat debris. The filtrate or ASL-L was kept in ice for further experiments.

### 2.4. Assay for Lipase Activity of ASL-L

*ρ*-nitrophenyl palmitate (*ρ*-NPP) was used as a substrate to determine lipase activity. Two solutions, namely solution A and solution B, were prepared. Solution A contained 16.6 mM *ρ*-NPP in isopropanol and solution B contained 0.4% (*w*/*v*) gum Arabic, 0.6% (*w*/*v*) triton X-100 in 50 mM tris HCl buffer (pH 7.5). Solution A (100 µL) and solution B (2.8 mL) were mixed and incubated in a water bath at 37 °C for 5 min. Subsequently, the substrate mixture was added with ASL-L (100 µL) and incubated at 37 °C for 10 min. The hydrolytic activity of ASL-L was measured at 410 nm. Nitrophenol (1–10 mM) was used to prepare the standard curve. One unit of ASL-L was defined as the required amount of enzyme to release 1 mg of nitrophenol/min under the assay condition [23,24].

### 2.5. ASL-L-Based Transesterification of CBF to Enrich STEO with Medium-Chain Fatty Acids (MCFAs)

The substrate mixture for transesterification was prepared following the method documented by Mogi et al. [25]. STEO and CBF were heated using a heat block at 50 °C for 30 min. Enzymatic solvent-free transesterification was conducted using 15 mL conical tubes on a Multi-Speed Vortex (MSV-3500, Biosan, Riga, Latvia). The substrate mixture was prepared by mixing 0.5 g of STEO with 1.5 g of CBF. Then, the mixture was added with 0.5 mL of ASL-L (50 U). The reaction mixture was placed on a high-speed vortex at 3000 rpm at 30 °C ± 2 °C for 24 h. Samples were collected after 24 h of reaction and subjected to GC-FID.

### 2.6. Optimization of ASL-L-Based Transesterification of CBF to Enrich STEO with MCFAs

#### 2.6.1. Effect of Time on STEO Enrichment with MCFAs

Transesterification was performed as mentioned in Section 2.5. The samples were collected at 0 h, 12 h, 24 h, 36 h, 48 h, 60 h, and 72 h and were subjected to GC-FID analysis.

#### 2.6.2. Effect of ASL-L Concentration on STEO Enrichment with MCFAs

ASL-L at different concentrations (50–500 U) was added to the reaction mixture and transesterification was conducted under the selected conditions with optimal time. Samples were collected after the reaction was accomplished and analyzed with GC-FID. The ASL-L concentration yielding a higher incorporation of medium-chain and n-3 fatty acids was chosen for further investigation.

#### 2.6.3. Effect of CBF/STEO Ratio on STEO Enrichment with MCFAs

CBF and STEO at varying ratios (2:1, 3:1, 4:1, 5:1, and 6:1 (*w*/*w*)) were used to prepare the substrate mixture. The transesterification was then conducted with the selected ASL-L concentration at the optimum ASL-L unit and reaction time. The products were analyzed with GC-FID and the ratio showing the maximum incorporation of MCFAs with intact n-3 fatty acids was selected for further investigations.

### 2.7. Analysis of Structured Lipid

#### 2.7.1. Gas Chromatography

The fatty acid profile of M-STEO in comparison with CBF and STEO was evaluated with gas chromatography connected to the flame ionization detector (GC-FID) (GC-FID; 7890B, Agilent, Santa Clara, CA, USA) [23,26]. The following GC conditions were followed: the flow rate of helium gas was 1.0 mL/min, the split ratio was set at 50:1, the detector temperature was 300 °C, and the injector temperature was set to 290 °C with the flow rate of 25 mL/min. The temperature of the GC was initially set at 210 °C and the holding time was set at 12 min, increased to 250 °C at 20 °C/min, and held for 8 min. The hydrogen flow was adjusted to 30 mL/min, while maintaining zero airflow at 300 mL/min for ignition purposes [23].

After transesterification, the samples were centrifuged at 8000× *g* for 5 min. The oil fraction was separated into hexane and subsequently, hexane was removed with nitrogen gas flash in a fume hood. Then, saponification was performed by adding 2M methanolic NaOH to 10 mg of oil dissolved in hexane. Samples were placed in a water bath at 50 °C for 5 min and stirred vigorously. The residual fractions were further treated with methanolic HCl to prepare the fatty acid methyl esters (FAME) of the samples [19]. After GC-FID analysis of the FAME, the individual fatty acids were quantified based on the peak area and reported as the percentage of total fatty acids [26].

#### 2.7.2. Lipid Profiles

##### Thin-Layer Chromatography–Flame Ionization Detector (TLC–FID)

The lipid compositions of samples including TAG, DAG, MAG, and fatty acids were determined with a TLC–FID. The chromarod S III; coated with silica gel, was dipped in boric acid (3%; *w*/*v*) for 5 min and dried. Thereafter, the rod was rescanned with a TLC–FID analyzer. Approximately, 1 μL of sample was spotted on the rod, and a mixture of benzene:chloroform:acetic acid at 52:20:0.7 (v/v/v) was used for separation. The rods were then dried at 105 °C for 5 min and were subjected to a flame ionization detector. The analyses were performed under the following conditions: an H_2_ flow rate of 160 mL/min, an airflow rate of 2000 mL/min, and a scanning speed of 30 sec/scan. The retention time of the lipid peaks in the FAME standards Supelco 37 Component FAME Mix C4–C24, (Merck, Darmstadt, Germany) was recorded. The peak areas were quantitatively determined and expressed as a percentage of the total lipid in the samples (AOAC, 1999).

##### ^1^H-NMR

The lipid profiles of M-STEO, CBF, and STEO were also determined using ^1^H-NMR following the method reported by Baloch et al. [20]. The transesterified oil was extracted using hexane and then the hexane was removed with nitrogen gas in a fume hood. M-STEO, CBF, and STEO (200 mg) were mixed with 400 µL of deuterated chloroform containing tetramethylsilane (TMS) as an internal reference. A Bruker Avance 400 spectrometer, which was operated at 500 MHz, was employed to determine the ^1^H-NMR spectra. The percentage of acyl groups (AG%) was determined using the following equation documented by Nieva-Echevarría et al. [27]:

AG_TG% = 100 (3NTG)/NTAG + FA(1)

AG_(1,2-DG)% = 100 (2N1,2-DG)/NTAG + FA(2)

AG_(1,3-DG)% = 100 (2N1,3-DG)/NTAG + FA(3)

AG_(2-MG)% = 100N2-MG (2N1,3-DG)/NTAG + FA(4)

AG_(1-MG)% = 100N1-MG (2N1,3-DG)/NTAG + FA(5)

FA% = 100NFA (2N1,3-DG)/NTAG + FA(6)

where AG denotes the acyl groups (%) relating to each type of glyceride in the samples, N denotes the amount of moles of different glycerides in the sample, and NTAG + FA represents the total moles of acyl groups and fatty acids in samples.

##### ^13^C NMR

The positional distributions of fatty acids in M-STEO, CBF, and STEO were further investigated with ^13^C NMR following the previous protocols [28,29,30]. After transesterification, the samples were subjected to the Brucker Avance 500 MHz spectrometer. The quantitative ^13^C NMR spectra of all the samples were determined under the constant decoupling of protons at 24 °C. Furthermore, 200 mg samples were prepared in 700 μL of CDCl3 and subjected to ^13^C NMR. All experiments were carried out in duplicates. The TopSpin 4.0.3 software (Bruker BioSpin, Rheinstetten, Germany) was used to evaluate the changes in the ^1^H-NMR and ^13^C-NMR patterns of CBF and STEO, and their esterified product (M-STEO).

### 2.8. Statistical Analysis

Data are represented as means ± standard deviations (SD). All experiments and analyses were done in triplicate. Comparison of experimental groups was determined using one- and two-way ANOVA followed by Tukey post hoc tests. All statistical analyses were performed using Prism version 8.0.1 (GraphPad Software, Inc., San Diego, CA, USA).

## 3. Results and Discussion

### 3.1. Transesterification of STEO for Enrichment with MCFAs in the Presence of CBF

Short- and medium-chain fatty acids are important contents of CBF [20,30], but they were not found in skipjack tuna eyeball oil (STEO) [15,19]. Nevertheless, STEO was abundant in long-chain n-3 fatty acids [15]. Therefore, STEO and CBF were subjected to lipase-mediated transesterification to acquire a modified STEO (M-STEO) rich in both MCFAs and PUFAs. After transesterification, MCFAs were found to be significantly incorporated in M-STEO (Table 1). The lauric acid (C12:0) content in M-STEO was 4.09%, capric acid (C10:0) was 1.82%, and caprylic acid (C8:0) was 2.05%. However, all these MCFAs were not found in the original STEO (Table 1). Butyric acid (C4:0), a short-chain fatty acid (SCFA), was 3.2% in M-STEO. Those fatty acids were mostly from CBF, which was incorporated into STEO after transesterification. It was worth noting that ASL-L could also retain the major long-chain saturated fatty acids including myristic acid (C14:0), pentadecanoic acid (C15:0), palmitic acid (C16:0), and stearic acid (C18:0) into M-STEO. All these SFAs were originally found in STEO as well as in CBF (Table 1).

Interestingly, ASL-L-mediated transesterification could incorporate MCFAs into STEO while retaining the major PUFAs found in STEO. In comparison with STEO, ASL-L-based transesterification yielded 64% of EPA and 35% of DHA in M-STEO after 24 h of transesterification. Moreover, the major PUFAs present in STEO were highly preserved by ASL-L and retained in the resulting M-STEO (Table 1).

ASL-L could preserve the major MUFAs in the M-STEO. Oleic acid (C18:1), which was one of the abundant MUFAs found in CBF (20%) and STEO (12.5%), constituted 19% of M-STEO after transesterification for 24 h. Similarly, the palmitoleic acid contents in CBF and STEO were 1.9% and 6.6%, respectively, which was 3.4% in M-STEO after 24 h of ASL-L-based transesterification (Table 1).

The produced M-STEO showed similar fatty acid components to those of IFM [9] (Table 2). The transesterification conditions for M-STEO production were further optimized. Since lipases from marine sources are more likely to hydrolyze or esterify polyunsaturated and monounsaturated fatty acids in marine animal lipids than lipases from other sources including microbial lipases [17,18], it would be more promising to use fish lipases to produce sLPs rich in n-3 fatty acids.

### 3.2. Optimization of ASL-L-Based Transesterification for the Enrichment of STEO with MCFAs in the Presence of CBF

Numerous parameters, such as reaction time, enzyme concentration, and CBF/STEO ratios were studied to optimize the enrichment of M-STEO with MCFs and PUFAs.

#### 3.2.1. Effect of Reaction Time on STEO Enrichment with MCFAs

**Figure 1:**
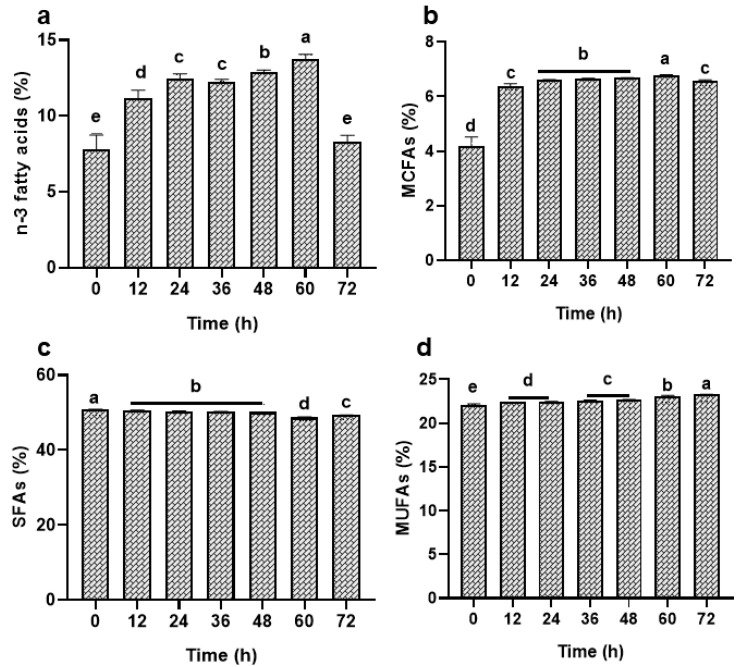
Effect of reaction time on the transesterification of commercial butterfat and STEO for the incorporation of (**a**) n-3 fatty acids, (**b**) medium-chain fatty acids, (**c**) saturated fatty acids, and (**d**) monounsaturated fatty acids in M-STEO. Bars represent the standard deviation (n = 3). Lowercase letters on the bars indicate significant differences (*p* < 0.05).

The STEO enrichment with MCFAs was performed up to 72 h to allow ASL-L to incorporate the maximum MCFAs in STEO. At 60 h, ASL-L rendered the highest levels of PUFAs, MCFAs, and MUFAs in the resulting M-STEO while having the lowest total SFAs (*p* < 0.05) (Figure 1). ASL-L could add 3.42% butyric acid (C4:0), 2.11% caproic acid (C6:0), 1.45% caprylic acid (C8:0), 2.73% capric acid (C10:0), and 3.4% lauric acid (C12:0) to the newly formed M-STEO after 60 h (Table 3). Reaction time for transesterification generally plays an important role in obtaining the reaction equilibrium toward the catalytic performance [23]. The rate at which the reaction proceeds is influenced by various factors such as temperature, enzyme concentration, reactant concentration, and mixing intensity [31]. However, even under optimal conditions, transesterification still requires sufficient time to generate the desired ester products [32].

On the other hand, ASL-L also had the capability to integrate long-chain saturated fatty acids (SFAs) into M-STEO. Following a 60 h transesterification process, ASL-L yielded M-STEO containing 10.6% myristic acid (C14:0), 1.7% pentadecanoic acid (C15:0), 31.70% palmitic acid (C16:0), 1.1% heptadecanoic acid (C17:0), 8.65% stearic acid (C18:0), 0.7% arachidic acid (C20:0), and 1.63% heneicosanoic acid (C21:0) (Table 3).

ASL-L could add n-3 long-chain PUFAs to M-STEO including 0.3% linolenic acid (C18:3), 2.84% EPA (C20:5), and 13.2% DHA (C22:6) after 60 h (Table 3). Through ASL-L-mediated transesterification of CBF and STEO, several n-6 long-chain PUFAs were also integrated into M-STEO. After a continuous transesterification for 60 h, the final product, i.e., M-STEO, was enriched with 1.3% linoleic acid (C18:2), 0.63% eicosadienoic acid (C20:2), and 0.28% linolenic acid (C20:3) (Table 3). Moreover, ASL-L added 0.54% myristoleic acid (C14:1), 2.63% palmitoleic (C16:1), 0.33% heptadecanoic acid (C17:1), 2.5% elaidic acid (C18:1), and 20.5% oleic acid (C18:1) to M-STEO, which were higher levels than those present in the substrate mixture (Table 3).

In the presence of certain regioselective lipases such as 1,3 regiospecific or 2 regiospecific lipase, the corresponding fatty acids are replaced by short- and medium-chain fatty acids [33]. The source of the lipase, the substrate mixture, the final product, as well as the conditions of the reaction could affect the time taken to attain the optimal catalytic capacity for lipase-mediated transesterification [23]. *Candida rugosa* lipase reached the optimum transesterification at 48 h to convert jatropha seed oil to biodiesel [34], while the immobilized *Candida rugosa* lipase took 120 h to synthesize ethyl butyrate at the optimum level [35]. Similarly, ASL-L could hydrolyze the lipids in the Asian seabass skin after 2 h of hydrolysis [22], whereas it showed optimum transesterification after 60 h (Figure 1).

#### 3.2.2. Effect of ASL-L Concentrations on STEO Enrichment with MCFAs

**Figure 2:**
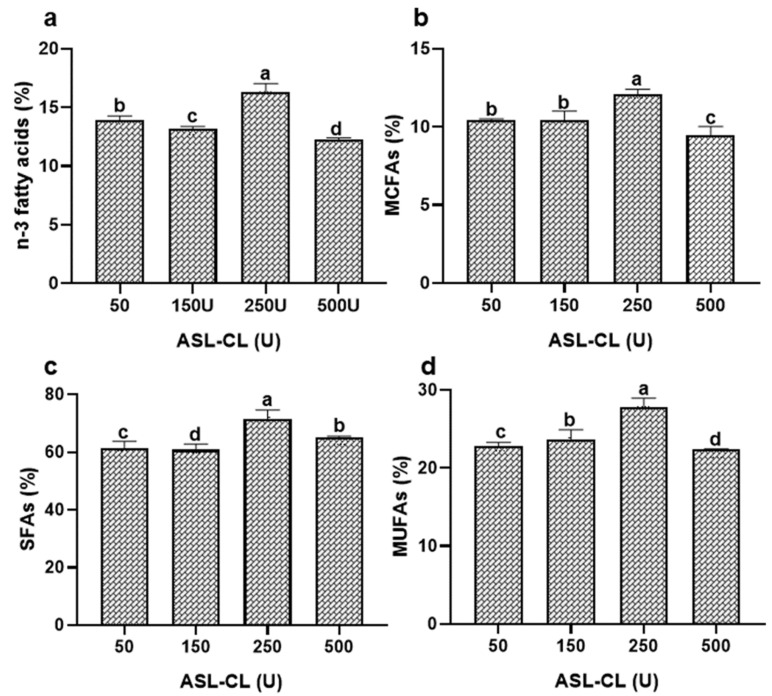
Effect of various ASL-L units on the transesterification of commercial butterfat and STEO for the incorporation of (**a**) n-3 fatty acids, (**b**) medium-chain fatty acids, (**c**) saturated fatty acids, and (**d**) monounsaturated fatty acids in M-STEO. Bars represent the standard deviation (n = 3). Lowercase letters on the bars indicate significant differences (*p* < 0.05).

Enzyme and substrate concentrations are crucial for optimizing transesterification [32]. Therefore, several units of ASL-L, including 50, 150, 250, and 500, were employed for the transesterification process. The amount of all kinds of fatty acids including SFAs, MCFAs, MUFAs, and PUFAs in the resulting M-STEO increased with increasing the concentration of ASL-L from 5 to 250 U (Figure 2). ASL-L was found to actively perform transesterification at its highest rate at 250 U. However, when the ASL-L concentration was increased to 500 U, the rate of transesterification was reduced, thus lowering the rate of fatty acid incorporation into STEO (Figure 2). Hydrolysis might have contributed to this reduction in fatty acid content. The abrupt shift in the enzyme-to-substrate ratio may have caused the equilibrium of the reaction to favor hydrolysis over transesterification, thus releasing more free fatty acids [36,37,38].

At 250 U, 12% of SCFAs and MCFAs were found to be incorporated in M-STEO. Butyric acid (C4:0) of 3.45%, caproic acid (C6:0) of 2.45%, caprylic acid (C8:0) of 2.01%, capric acid of 2.94%, and lauric acid (C12:0) of 4.03% were observed in M-STEO. Among the long-chain SFAs, 9.51% of myristic acid (C14:0), 1.72% of pentadecanoic acid (C15:0), 26.8% of palmitic acid (C16:0), 1.3% of heptadecanoic acid (C17:0), 8.13% of stearic acid (C18:0), 0.65% of arachidic acid (C20:0), and 1.55% of heneicosanoic acid (C21:0) were added to M-STEO by ASL-L-mediated transesterification (Table 3).

ASL-L also incorporated n-3 and n-6 long-chain PUFAs in M-STEO, comprising 1.57% linoleic acid (C18:2), 0.37% linolenic acid (C18:3), 2.2% EPA (C20:5), and 12.51% DHA (C22:6) at 250 U (Table 3). Additionally, among the MUFAs present in the substrate mixture, ASL-L incorporated 0.81% myristoleic acid (C14:1), 3.34% palmitoleic acid (C16:1), 0.84% heptadecanoic acid (C17:1), 0.86% elaidic acid (C18:1), and 15.3% oleic acid (C18:1) to M-STEO (Table 3). Similar effects of enzyme concentration were reported when Lipozyme RM and Lipozyme TL were used for the interesterification of coconut oil and catfish oil to produce modified lipids containing both MCFAs and PUFAs at their glycerol backbones [39].

#### 3.2.3. Effect of CBF/STEO Ratio on STEO Enrichment with MCFAs in the Presence of CBF

**Figure 3:**
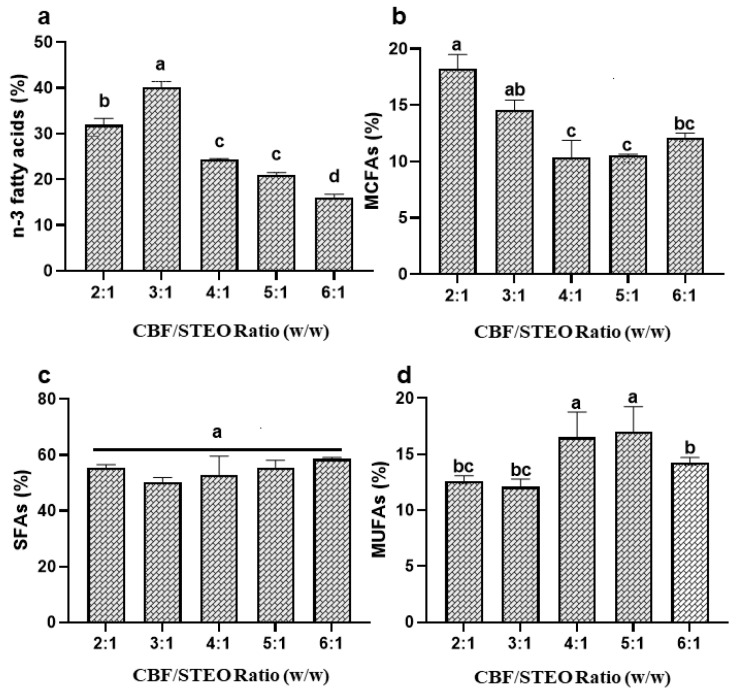
Effect of CBF/STEO ratios on the transesterification of commercial butterfat and STEO for the incorporation of (**a**) n-3 fatty acids, (**b**) medium-chain fatty acids, (**c**) saturated fatty acids, and (**d**) monounsaturated fatty acids in M-STEO. Bars represent the standard deviation (n = 3). Lowercase letters on the bars indicate significant differences (*p* < 0.05).

The optimum ratio of acyl donors and acyl acceptors is an important factor for reactions such as transesterification and esterification to produce certain desired structured fatty acids [38]. However, the transesterification reaction starts with liberating the fatty acids from the respective ester bonds and resulting in a pool of fatty acids serving as acyl donors, and acyl acceptors in the reaction mixture in the form of FFAs, MAGs, and DAGs, which are further esterified into TAGs with different types and rearrangements of fatty acids at different sn-positions [11]. The tuna eyeball oil contained 85–95% TAGs and CBF had around 96% TAGs with different fatty acids [19,21]. Therefore, STEO and CBF were added at different ratios (*w*/*w*) in the reaction mixture to find the optimum ratio. At a CBF/STEO ratio of 3:1, 3.56% butyric acid (C4:0), 3.8% caproic acid (C6:0), 1.1% caprylic acid (C8:0), 1.4% capric acid (C10:0), and 3.9% lauric acid (C12:0) were incorporated into M-STEO (Table 3). ASL-L showed maximum incorporation (40.2%; *p* < 0.05) of n-3 fatty acids when a CBF/STEO ratio of 3:1 (*w*/*w*) was used. Nevertheless, a further increase in CBF proportion in the reaction mixture reduced the incorporation of n-3 fatty acids (Figure 3a). ASL-L could add 1.96% linoleic acid (C18:2), 0.70% linolenic acid (C18:3), 6.38% EPA (C20:5), and 17.9% DHA (C22:6) at a CBF/STEO ratio of 3:1 (Table 3). Similar results were observed when immobilized lipases from *Aspergillus niger* and *Rhizopus javanicus* and Lipozyme 435 were applied for the incorporation of n-3 fatty acids in the soybean oil and both lipases showed maximum n-3 fatty acid incorporation at 3:1 (acyl donor:acyl acceptor) ratio [39,40].

At higher CBF proportions such as 4:1 and 5:1 (CBF: STEO; *w*/*w*), the ASL-L preferred to incorporate MUFAs at 16.5% and 17.0%, respectively, but caused reduced n-3 fatty acid and MCFA insertion in the glycerol backbone (Figure 3b,d). At a ratio of 3:1 (CBF: STEO), ASL-L amalgamated 0.46% myristoleic acid (C14:1), 2.7% palmitoleic acid (C16:1), 0.51% heptadecanoic acid (C17:1), 0.66% elaidic acid (C18:1), and 15.1% oleic acid (C18:1) to the resulting M-STEO. The optimal insertion of MCFAs (18.2%) was observed at a CBF/STEO ratio of 2:1, followed by 3:1 (14.6%), and a further increase in the CBF proportion reduced the number MCFAs in the M-STEO (Figure 3b). Similarly, *Rhizomucor miehei* and *Thermomyces lanuginosa* lipases showed a high incorporation of medium-chain fatty acids into canola oil at a substrate ratio of 3:1 [41]. Nonetheless, there were no significant changes in the total SFA concentration (*p* > 0.05), regardless of changes in CBF/STEO ratios (Figure 3c).

### 3.3. Lipid Profiles and Fatty Acid Distribution

#### 3.3.1. Quantification of Glycerides by TLC–FID

**Figure 4:**
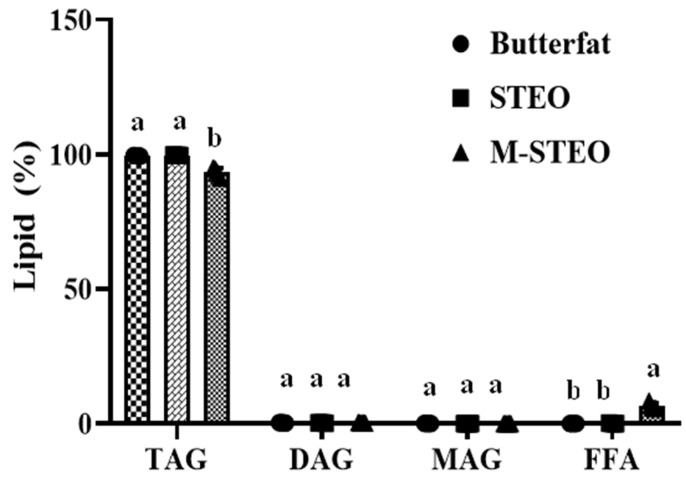
Lipid profile of butterfat, STEO, and M-STEO analyzed by TLC–FID. Bars represent the standard deviation (n = 3). Lowercase letters on the bars within the same type of lipid indicate significant differences (*p* < 0.05).

The number of glycerides including TAG, DAG, MAG, and free fatty acids was measured by TLC–FID. There were 99.58% TAG, 0.32% DAG, 0.05% MAG, and 0.05% free fatty acids in CBF. STEO contained 99.68% TAG, 0.20% DAG, 0.02% MAG, and 0.11% free fatty acid (Figure 4). M-STEO produced by ASL-L via the transesterification of CBF and STEO under optimum conditions contained 93.56% TAG, 0.31% DAG, 0.02% MAG, and 6.5% free fatty acids (Figure 4). The findings demonstrated that ASL-L could redistribute the fatty acids on the glycerol backbone without affecting the natural lipid profile of the reactants and that the reaction conditions were favorable for esterification rather than hydrolysis. The original numbers of TAG, DAG, and MAG in CBF and STEO also remained unabated in the newly formed M-STEO. He et al. [42] applied Novozym 435, Lipozyme 435, and Lipozyme TL-IM lipases to produce human milk fat substitutes with the algal oils extracted from *Nannochloropsis oculata* and *Isochrysis galbana*. The TAG levels of 34% remained unchanged, compared to the original 38–39% TAG content in *Nannochloropsis oculate* oil. However, in the present study, 93.8% of TAGs remained intact in M-STEO. The concentration of fatty acids and their glycerides category in human milk depends on ethnicity, region, lactation stage, maternal diet, and mammary gland synthesis ability [43]. However, human milk mainly consists of TAGs (98–99%) with a small amount of DAGs and MAGs all over the lactation period [44]. Interestingly, the TAG concentration in M-STEO was 93.56% with negligible traces of DAGs and MAGs. Hence, M-STEO could be utilized to formulate milk with lipids mimicking that of human milk.

#### 3.3.2. ^1^H-NMR

**Figure 5:**
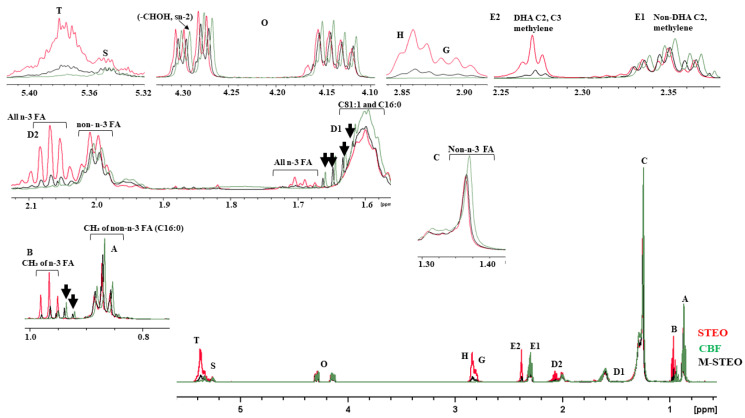
^1^H NMR spectrum of STEO (red lines), CBF (green lines), and M-STEO (black lines) after ASL-L-based transesterification of STEO and CBF under optimum conditions. The expanded inset shows the detailed regions of interest.

Figure 5 displays the ^1^H-NMR spectra of CBF, STEO, and M-STEO and their extended regions. The changes in peak intensities (A, B, D2, D1, E1, E2, O, S, T) showed that remarkable modifications in lipid composition occurred in the glycerol backbone due to the transesterification reaction. The ^1^H-NMR spectra representing n-3 fatty acids were found in STEO and M-STEO, but they were not seen in the ^1^H-NMR spectrum of CBF (Figure 5 B, D2, E2). The conventional proton signals corresponding to acyl groups (B, D2, D1, E1, E2, T) were present in all samples including the signal representing the glycerol backbone of the TAG at the O and S locations (Figure 5). On the other hand, signals located at the D2 and E2 positions were associated with docosahexaenoic acyl groups and eicosapentaenoic together with arachidonic acyl groups, respectively [27]. Major non-n-3 fatty acid signals were observed in CBF, STEO, and M-STEO (C, D1, O, D2, E1), showing the major saturated TAGs. The expended spectra in Figure 5 H and G show the allylic proton in the samples. The results indicated an intact TAG structure and minimum loss of fatty acids during the transesterification reaction. Furthermore, the ^1^H-NMR spectra of CBF and M-STEO showed some unidentified peaks that were absent in STEO (Figure 5 arrows). These peaks might be associated with the short- and medium-chain fatty acids from CBF, which might be incorporated into M-STEO via transesterification by ASL-L. The chemical shift that occurred in M-STEO, compared to that of CBF and STEO was due to the unabated fatty acid profile of the M-STEO after transesterification. M-STEO contained intact TAG with minimum DAG, MAG, and free fatty acids. Such a negligible shift in the acyl groups confirmed the structural intactness of the lipids from CBF and STEO after their optimal transesterification using ASL-L to produce the structured fatty acid, M-STEO.

### 3.4. Positional Distribution of Major Fatty Acid

**Figure 6:**
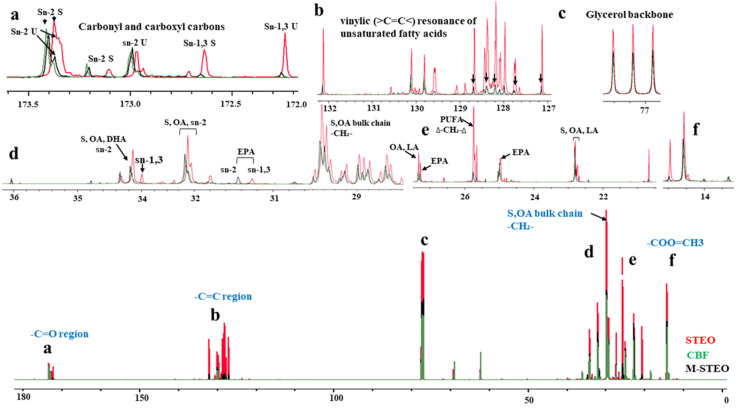
^13^C NMR spectrum of STEO (red lines), CBF (green lines), and M-STEO (black lines) after ASL-L-based optimum transesterification. The expanded inset spectra show the details of the region of interest. The expanded peaks represent the following (**a**) carbonyl and carboxylic carbons of SFAs and UFAs at sn-1,3, (**b**) vinylic and allylic carbon resonances of saturated and unsaturated fatty acids, (**c**) the glycerol backbone of the fatty acids, (**d**–**f**) the aliphatic carbon resonances of saturated, unsaturated carbons and carbons from free fatty acids as well.

Although, the fatty acid contents of human milk account for 3.0–5.0% of the total milk composition, they contribute to more than 50% of the required energy for infant growth and development [42]. The positional distribution of certain fatty acids on the glycerol backbone is very important for the nutritional capacities of the target fatty acids [29]. Human milk contains 25–30% palmitic acid and 50–70% of which is distributed at the sn-2 position of the glycerol backbone [9,45]. Vinylic and allylic carbon resonances are the differentiating characteristic of UFAs and SFAs in ^13^C NMR and they can further help in the characterization and classification of the UFAs and SFAs [46]. The major carbonyl and carboxylic carbons of SFAs and UFAs at sn-2 appear at approximately 173 ppm whereas the carbonyl and carboxylic carbons of SFAs and UFAs at sn-1,3 are represented by the ^13^C NMR peaks at 172 ppm [47]. The intact region of the carboxylic spectra contains four pairs of peaks with 0.38 and 0.39 ppm chemical shift differences [46]. These spectral regions showed DHA and EPA along with other unsaturated fatty acids, as well as palmitic acid and some other saturated fatty acids (Figure 6a). The major vinylic carbons (carbons that have double bonds) that represent unsaturated carbons of fatty acids appeared at 124–134 ppm. The ^13^C NMR signals obtained from STEO showed the highest peak intensity, followed by M-STEO, whereas CBF showed minimum peaks in this region (Figure 6a).

The results indicated that ASL-L could incorporate the major UFAs in M-STEO. The major positional distributions of DHA, EPA, and saturated fatty acids were observed at the sn-2 position of the glycerol backbone, which was in line with the observation by Siddiqui et al. [45]. The modified STEO (M-STEO) was highly enriched with short- and medium-chain SFAs including 3.56% butyric acid (C4:0), 3.8% caproic acid (C6:0), 1.1% caprylic acid (C8:0), 1.4% capric acid (C10:0), and 3.9% lauric acid (C12:0) (Table 3). The saturated fatty acids at the sn-2 position in Figure 6 at 173.3 ppm of the ^13^C NMR spectra indicated the incorporated medium-chain TAG in M-STEO which was absent in the original tuna eyeball oil. The peaks that represent the glycerol backbone and aliphatic carbons are at 60–72 ppm and 10–35 ppm, respectively, [19] showing intact intensity among the sample (Figure 6c), confirming the intact presence of the glycerol backbone after transesterification. The aliphatic carbon resonances representing both saturated and unsaturated fatty acids revealed that the major SFAs and UFAs in M-STEO were located at the sn-2 position (Figure 6d). Among the long-chain saturated fatty acids, palmitic acid was around 25–30% in M-STEO produced under optimum conditions, whereas the positional distribution estimated from the ^13^C NMR peaks showed that the major distribution of saturated and unsaturated fatty acids in M-STEO was at the sn-2 position [29,46]. Therefore, M-STEO not only resembled HMFAs in the context of fatty acid contents but it also shared a very close positional distribution with that of HMFAs.

## 4. Conclusions

Asian seabass liver lipase could incorporate essential fatty acids including long-chain n-3 and n-6 fatty acids from tuna eyeball oil, as well as short- and medium-chain fatty acids from commercial butterfat to produce structured lipids. The incorporation of fatty acids into newly formed M-STEO was confirmed by GC-FID. The lipid profiles including tri-, di-, and mono-glycerides were determined by TLC–FID and ^1^H-NMR. The positional distribution was studied by ^13^C NMR. The newly formed oil M-STEO was highly enriched with short-, medium-, and long-chain saturated and unsaturated fatty acids. Furthermore, the positional distribution of the newly formed structured fatty acid (M-STEO) closely resembled the positional distribution of human milk fatty acids. Further steps including the immobilization of ASL-L on certain carriers and the replacement of commercial butterfat with that of dairy waste fat would further improve the efficient use of enzymes as well as reduce the overall production cost. Moreover, advanced studies on M-STEO would be helpful for future endeavors to develop structured lipids for infant formula milk that could closely mimic human milk’s fatty acid composition.

## Figures and Tables

**Table 1:** Fatty acid profile of butterfat, STEO, and the ASL-L-modified STEO (M-STEO).

	Saturated Fatty Acids (%)		
Fatty Acids	Butterfat	STEO	M-STEO
Butyric acid (C4:0)	1.42 ± 0.03b	NA	3.20 ± 0.50
Caproic acid (C6:0)	1.41 ± 0.02b	NA	2.50 ± 0.30
Caprylic acid (C8:0)	1.05 ± 0.01b	NA	2.05 ± 0.25
Capric acid (C10:0)	2.70 ± 0.10a	NA	1.82 ± 0.08
Lauric acid (C12:0)	4.73 ± 0.33a	NA	4.09 ± 0.50
Tridecanoic (C13:0)	0.20 ± 0.01a	0.06 ± 0.00c	0.13 ± 0.00b
Myristic acid (C14:0)	12.5 ± 0.50a	3.91 ± 0.40c	9.43 ± 0.80
Pentadecanoic acid (C15:0)	1.40 ± 0.06a	1.51 ± 0.10a	1.70 ± 0.09a
Palmitic acid (C16:0)	33.60 ± 2.13a	22.7 ± 1.52c	25.10 ± 0.60
Heptadecanoic acid (C17:0)	0.70 ± 0.00b	0.93 ± 0.04a	1.60 ± 0.10b
Stearic acid (C18:0)	10.4 ± 0.60a	5.00 ± 0.23c	7.20 ± 0.09
Arachidic acid (C20:0)	0.10 ± 0.01a	0.50 ± 0.12a	0.30 ± 0.00
Heneicosanoic acid (C21:0)	1.40 ± 0.05a	0.41 ± 0.01c	1.16 ± 0.02b
Docosanoic acid (C22:0)	NA	1.10 ± 0.06a	0.30 ± 0.08b
Tricosanoic (C23:0)	NA	2.01 ± 0.15a	1.03 ± 0.01b
	**Monounsaturated Fatty Acids (%)**		
Myristoleic acid (C14:1n-5)	1.20 ± 0.02a	0.03± 0.00	1.30 ± 0.01
Palmitoleic acid (C16:1n-7	1.90 ± 0.05c	1.2 ± 0.02	3.40 ± 0.20
Heptadecanoic acid (C17:1)	0.36 ± 0.01b	0.93 ± 0.04a	0.90 ± 0.05a
Oleic acid (C18:1n-9)	20.0 ± 1.31a	12.5 ± 0.90b	14.0 ± 0.40a
Elaidic acid (C18:1)	3.40 ± 0.21a	0.11 ± 0.03c	2.20 ± 0.06b
Nervonic acid (C24:1)	NA	0.53 ± 0.01a	0.23 ± 0.02a
	**n-3 Polyunsaturated Fatty Acids (n-3 PUFAs) (%)**		
α-Linolenic acid (C18:3n-3)	0.70 ± 0.00a	1.11 ± 0.01	0.9 ± 0.00
Eicosapentaenoic acid (C20:5n-3)	NA	3.61 ± 0.30a	2.32 ± 0.24
Clupanodonic acid (C22:5n-3)	NA	-	-
Docosahexaenoic acid (C22:6n-3)	Na	33.10 ± 3.2a	11.5 ± 1.50b
	**n-6 Polyunsaturated Fatty Acids (n-6 PUFAs) (%)**		
Linoleic acid (C18:2n-6)	1.10 ± 0.00a	1.50 ± 0.13a	1.54 ± 0.03a
Eicosadienoic acid (C20:2n-6)	NA	0.23 ± 0.03a	0.85 ± 0.04a
Dihomo-γ-Linolenic acid (C20:3n-6)	NA	0.60 ± 0.02a	0.5 ± 0.04b

Means ± SD (n = 3). NA: Not Available. Different lowercase letters in the same row indicate a significant difference (*p* < 0.05).

**Table 2:** Comparison of fatty acid contents found in human milk, infant formula milk, and the ASL-L-modified STEO (M-STEO).

	Saturated Fatty Acids (%)		
Fatty Acids	Human Milk Fat [31]	Formula Milk Fat [9]	M-STEO
Butyric acid (C4:0)	-	1.75 ± 0.03	3.20 ± 0.50
Caproic acid (C6:0)	-	0.01 ± 0.01	2.50 ± 0.30
Caprylic acid (C8:0)	0.2–0.3	0.72 ± 0.04	2.05 ± 0.25
Capric acid (C10:0)	1.5–1.8	-	1.82 ± 0.08
Lauric acid (C12:0)	5.7–6.5	12.27 ± 0.01	4.09 ± 0.50
Myristic acid (C14:0)	6.5–7.1	4.71 ± 0.02	9.43 ± 0.80
Palmitic acid (C16:0)	21.7–22.7	27.45 ± 0.11	25.10 ± 0.60
Stearic acid (C18:0)	6.3–6.6	4.54 ± 0.04	7.20 ± 0.09
Arachidic acid (C20:0)	0.20–0.26	0.33 ± 0.02	0.30 ± 0.00
	**Monounsaturated Fatty Acids (%)**		
Myristoleic acid (C14:1n-5)	0.18–0.22	0.03± 0.00	1.30 ± 0.01
Palmitoleic acid (C16:1n-7	2.2–2.4	1.2 ± 0.02	3.40 ± 0.20
Oleic acid (C18:1n-9)	32.2–33.6	32.7 ± 0.13	14.0 ± 0.40
Vaccenic acid (C18:1n-7)	1.7–2.1	-	2.20 ± 0.06
Erucic acid (C22:1n-9)	0.10–0.12		-
	**n-3 Polyunsaturated Fatty Acids (n-3 PUFAs) (%)**		
α-Linolenic acid (C18:3n-3)	0.91–1.03	1.16 ± 0.01	0.9 ± 0.00
Eicosapentaenoic acid (C20:5n-3)	0.08–0.10	-	2.32 ± 0.24
Clupanodonic acid (C22:5n-3)	0.14–0.16	-	-
Docosahexaenoic acid (C22:6n-3)	0.28–0.34	-	11.5
	**n-6 Polyunsaturated Fatty Acids (n-6 PUFAs) (%)**		
Linoleic acid (C18:2n-6)	14.3–15.7	13.99 ± 0.06	1.54
γ-Linolenic acid (C18:3n-6)	0.14–0.20	-	-
Eicosadienoic acid (C20:2n-6)	0.35–0.41	-	0.85 ± 0.04
Dihomo-γ-Linolenic acid (C20:3n-6)	0.39–0.43	-	0.5 ± 0.04
Arachidonic acid (C20:4n-6)	0.45–0.51	-	-
Docosatetraenoic acid (C22:4n-6)	0.09–0.11	-	-
Adrenic acid (C22:5n-6)	0.06–0.10	-	-
Total SFAs	42.1	51.78	61.61
Total MUFAs	36.38	33.93	22.13
Total PUFAs	17.20	15.60	17.61
Total trans fats			

**Table 3:** Fatty acid composition of M-STEO produced by ASL-L for each step of optimization of transesterification.

	Saturated Fatty Acids (%)		
Fatty Acids	Step 1	Step 2	Step 3
Butyric acid (C4:0)	3.42 ± 0.27a	3.45 ± 0.23a	3.56 ± 0.41a
Caproic acid (C6:0)	2.11 ± 0.36b	2.45 ± 0.01b	3.8 ± 0.27a
Caprylic acid (C8:0)	1.45± 0.00b	2.01 ± 0.00a	1.1 ± 0.06b
Capric acid (C10:0)	2.73 ± 0.01a	2.94 ± 0.00a	1.4 ± 0.10b
Lauric acid (C12:0)	3.40± 0.38a	4.03 ± 0.04a	3.9 ± 0.01a
Myristic acid (C14:0)	10.6 ± 0.05a	9.51 ± 0.12a	7.6 ± 0.01b
Pentadecanoic acid (C15:0)	1.70 ± 0.00a	1.72 ± 0.00a	1.4 ± 0.08a
Palmitic acid (C16:0)	31.70 ± 2.16a	26.80 ± 1.30b	25.0 ± 0.21b
Heptadecanoic acid (C17:0)	1.10 ± 0.01a	1.30 ± 0.36a	1.60 ± 0. 10a
Stearic acid (C18:0)	8.65 ± 0.01a	8.13 ± 0.01b	1.61 ± 0.01c
Arachidic acid (C20:0)	0.70 ± 0.01a	0.65 ± 0.01a	0.46 ± 0.00b
Heneicosanoic acid (C21:0)	1.63 ± 0.05a	1.55 ± 0.01b	1.68 ± 0.02a
	**Monounsaturated Fatty Acids (%)**		
Myristoleic acid (C14:1n-5)	0.54 ± 0.02b	0.81 ± 0.00a	0.46 ± 0.03c
Palmitoleic acid (C16:1n-7	2.63 ± 0.05b	3.34 ± 0.14a	2.70 ± 0.22b
Heptadecanoic acid (C17:1)	0.33 ± 0.20b	0.84 ± 0.04a	0.51 ± 0.01b
Oleic acid (C18:1n-9)	20.5 ± 0.98a	15.3 ± 0.10b	15.1 ± 0.05b
Elaidic acid (C18:1)	2.50 ± 0.12a	0.86 ± 0.01b	0.66 ± 0.001c
	**n-3 Polyunsaturated Fatty Acids (n-3 PUFAs) (%)**		
α-Linolenic acid (C18:3n-3)	0.30 ± 0.00c	0.37 ± 0.00b	0.70 ± 0.01a
Eicosapentaenoic acid (C20:5n-3)	2.84 ± 0.03c	2.22 ± 0.00b	6.38 ± 0.2a
Docosahexaenoic acid (C22:6n-3)	13.2 ± 0.05b	12.51 ± 0.09c	17.9 ± 0.81a
	**n-6 Polyunsaturated Fatty Acids (n-6 PUFAs) (%)**		
Linoleic acid (C18:2n-6)	1.30 ± 0.00c	1.57 ± 0.02b	1.96 ± 0.04a
Eicosadienoic acid (C20:2n-6)	0.63 ± 0.03a	0.58 ± 0.00b	0.66 ± 0.00a
Dihomo-γ-Linolenic acid (C20:3n-6)	0.28 ± 0.00c	0.32 ± 0.00b	1.16 ± 0.00a

Step 1: Optimal time: 60 h (CBF/STEO: 1:3, ASL-L: 50 U, temperature: 30 °C). Step 2: Optimal ASL-L: 250 U (CBF/STEO: 1:3, time: 60 h, temperature: 30 °C). Step 3: Optimal CBF/STEO: 1:3 (ASL-L: 250 U, time: 60 h, temperature: 30 °C). Means ± SD (n = 3). Different lowercase letters in the same row indicate a significant difference (*p* < 0.05).

## Data Availability

Data is contained within the article.
